# Building a nuclear envelope at the end of mitosis: coordinating membrane reorganization, nuclear pore complex assembly, and chromatin de-condensation

**DOI:** 10.1007/s00412-012-0388-3

**Published:** 2012-10-27

**Authors:** Allana Schooley, Benjamin Vollmer, Wolfram Antonin

**Affiliations:** Friedrich Miescher Laboratory of the Max Planck Society, Tübingen, Germany

**Keywords:** Nuclear envelope formation, Nuclear pore complex assembly, Chromatin decondensation, Mitotic exit

## Abstract

The metazoan nucleus is disassembled and re-built at every mitotic cell division. The nuclear envelope, including nuclear pore complexes, breaks down at the beginning of mitosis to accommodate the capture of massively condensed chromosomes by the spindle apparatus. At the end of mitosis, a nuclear envelope is newly formed around each set of segregating and de-condensing chromatin. We review the current understanding of the membrane restructuring events involved in the formation of the nuclear membrane sheets of the envelope, the mechanisms governing nuclear pore complex assembly and integration in the nascent nuclear membranes, and the regulated coordination of these events with chromatin de-condensation.

## Introduction

A functional nucleus relies on the precise structural organization of its genome and the existence of an intact boundary that separates nuclear and cytoplasmic activities, the nuclear envelope (NE). These features are repeatedly established in the mitotically dividing cells of animals. While many lower eukaryotes employ closed or semi-closed mitosis, during which the NE remains at least partially intact (De Souza and Osmani 2009), the onset of mitosis in metazoan cells is marked by dramatic changes to nuclear architecture. Open mitosis requires the complete disassembly of the NE in order to form the mitotic spindle on condensed chromosomes. The consequence of this disassembly is the need to re-build the NE each time the cell divides.

**Fig. 1:**
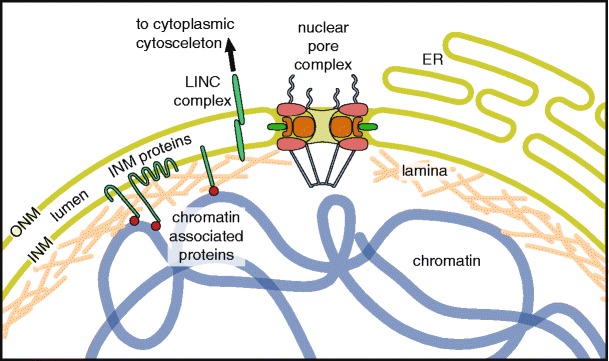
The vertebrate nuclear envelope. The two-membrane sheets of the nuclear envelope are separated by a lumenal space and are continuous with the bulk endoplasmic reticulum (*ER*) network. The outer nuclear membrane (*ONM*) and the inner nuclear membrane (*INM*) are fused at nuclear pores, where nuclear pore complexes are integrated to regulate bidirectional transport between the cytoplasm and the nucleoplasm. The INM is distinctly characterized by a set of integral membrane proteins that connect the nuclear envelope to chromatin by interacting directly or indirectly via chromatin-associated proteins and the nuclear lamina. The nuclear lamina is additionally connected to the cytoplasmic cytoskeleton by the interaction of LINC complex proteins of the ONM and INM across the NE lumen

The NE is composed of two concentric bilayers surrounding the chromatin: the outer nuclear membrane (ONM), which is continuous with the endoplasmic reticulum (ER), and the inner nuclear membrane (INM), separated from the ONM by a lumenal space (Fig. 1). These membranes are fused at sites of nuclear pore complex (NPC) integration. NPCs are large protein complexes that contribute to the diffusion barrier of the NE and act as a regulatory gateway for the bidirectional exchange of proteins, RNA, and ribonucleoprotein complexes between the nucleus and the cytoplasm (for review, see Wente and Rout 2010). While the outer membrane is biochemically and functionally similar to the ER, the inner membrane is distinctly characterized by a specific set of integral membrane proteins that establish connections to chromatin and, in metazoan cells, to the overlying nuclear lamina. The lamina is a meshwork of nucleus-specific intermediate filaments called lamins, which maintain the shape and mechanical stability of the nucleus (for review, see Gruenbaum et al. 2005; Shimi et al. 2010). The lamina is also indirectly connected to the cytoplasmic cytoskeleton via linker of nucleoskeleton and cytoskeleton (LINC) complexes that span the NE lumen (for review, see Burke 2012; Starr and Fridolfsson 2010).

Building a nucleus at the end of mitosis involves the complete reconstruction of nuclear membrane sheets and macromolecular NPCs on two sets of de-condensing chromosomes. Here, we review our current understanding of vertebrate NE reassembly as a coordinated process of membrane restructuring, NPC assembly, and chromatin de-condensation.

### Re-organizing the mitotic ER

The NE is a distinct domain of the ER, owing to direct and indirect interactions between NE-specific proteins and chromatin. During mitosis, these proteins are released from the disassembled lamina and the underlying chromatin, resulting in their redistribution throughout the ER and thus the absorption of the NE membranes in the ER network (Daigle et al. 2001; Ellenberg et al. 1997; Yang et al. 1997). At the end of mitosis, the dramatic reorganization of the mitotic ER gives rise to a new NE forming around each mass of segregating chromatin. The architectural starting point for this ER re-structuring is, however, a matter of debate. In addition to the NE, the entire ER network undergoes significant morphological changes during mitosis. According to two contradictory models, the interphase system of ER sheets and tubules is transformed into either a tubular ER or sheet-like network during mitosis.

**Fig. 2:**
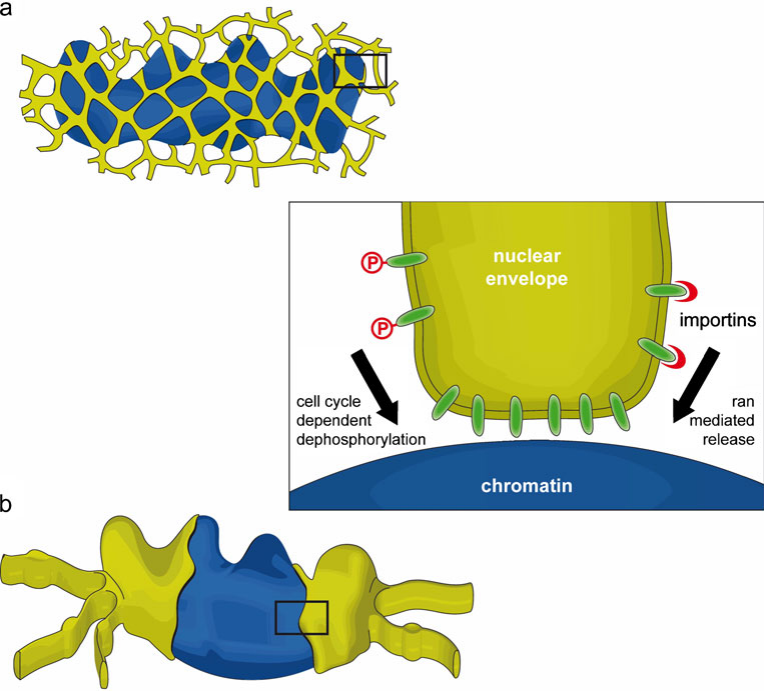
The nuclear envelope is constructed by the re-organization of the mitotic ER on the chromatin. Two models have been proposed to explain nuclear envelope formation based on the predominant organization of the ER during mitosis. In the first model (**a**), a tubular ER network contacts chromatin via tubule ends, which flatten and expand on the chromatin surface to give rise to nuclear envelope sheets. Alternatively, ER-derived membrane sheets initiate nuclear envelope formation by associating laterally with the chromatin mass and spreading around it (**b**). In both cases, the regulated recruitment of membrane proteins of the INM (*inset*, see “Regulating the recruitment of nuclear envelope membranes to chromatin”) mediates the accumulation of nuclear envelope-specific membranes and thus the establishment of this distinct ER subdomain

The mitotic ER has been observed as an exclusively tubular network (Puhka et al. 2007) and in vitro experiments suggest that an intact tubular ER is required for post-mitotic NE formation (Anderson and Hetzer 2007). This network is recruited via tubule ends that make first contact to the chromatin substrate and become immobilized (Fig. 2a). Subsequent flattening and lateral expansion of membranes on the chromatin surface is proposed to give rise to inner and ONM sheets. In further support of this model, ER tubules have been found to surround post-mitotic chromatin in vivo (Anderson and Hetzer 2008a). Overexpression of reticulons, proteins that shape the ER into tubules, delays NE formation while the depletion of reticulons by siRNA accelerates the formation of a closed NE. These experiments suggest that ER reshaping events, specifically those promoting membrane sheet formation from tubules, are crucial for NE assembly.

Recent live cell imaging and electron microscope tomography studies have provided evidence that NE re-assembly rather initiates from a cisternal, or sheet-like, mitotic ER (Lu et al. 2011) (Fig. 2b). During mitosis, the ER was found to consist almost entirely of extended cisternae, with the exception a few tubules contacting the mitotic spindle (Lu et al. 2009). Cisternal mitotic ER has also been observed in 3D reconstructions of light microscopy sections from *Caenorhabditis elegans* embryos (Poteryaev et al. 2005). The conservation of this ER structure in different cell types and organisms suggests that a sheet-like network could be a general feature of mitotic cells. NE assembly from extended cisternae is initiated by contact between ER sheets and chromatin (Lu et al. 2011). As membrane sheets enclose the chromatin they are organized into a NE-specific domain.

The organization of the interphase ER network varies between cell types and differentiation states (Voeltz et al. 2002). Similarly, the relative abundance of ER sheets and tubules is not the same in all mitotic cells (Puhka et al. 2012). Observations of entirely tubular or cisternal networks might therefore reflect extreme examples on a spectrum of possible mitotic ER arrangements. Assuming that the predominance of mitotic ER sheets and tubules varies between cell types, the question becomes: What is the morphology of the ER that contacts chromatin and gives rise to the sheets of the NE? The transformation of ER tubules into membrane sheets on the chromatin has not been directly visualized (Anderson and Hetzer 2008a). Reticulon-positive membrane tubules have been recorded around the post-mitotic chromatin mass in live cells but in this case the tubules dynamically contact chromatin and do not directly contribute to the NE (Lu and Kirchhausen 2012). It therefore seems likely that the conversion of tubules to cisternal sheets is a prerequisite for the stable association of future NE membranes with chromatin.

**Fig. 3:**
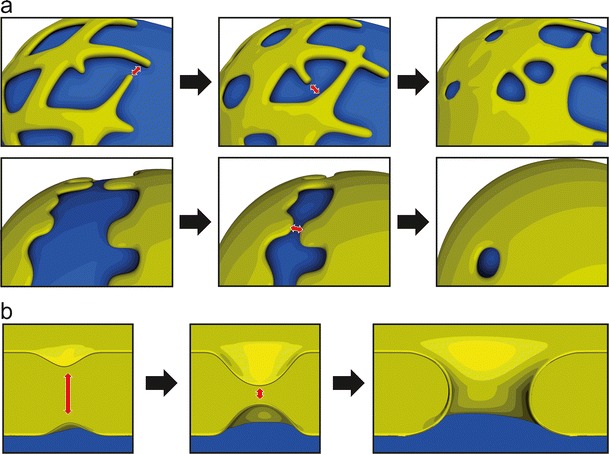
Membrane fusion is required for nuclear envelope formation. Cytoplasmic fusion between outgrowing ER-derived tubules (**a**, *upper*) or sheets (**a**, *lower*) is required for re-assembly of a nuclear envelope around the chromatin mass at the end of mitosis. A second type of fusion between the outer and INMs across the lumenal space is required to create a pore in the intact nuclear envelope (**b**) for the insertion of NPCs during interphase and possibly post-mitotically

Regardless of whether it is initiated by the outgrowth of ER tubules or from cisternal ER sheets, the complete enclosure of chromatin by the NE requires membrane fusion (Fig. 3a). As a subdomain of the ER, it is plausible that the NE employs the ER membrane fusion machinery to achieve this task. Many of the cellular membrane fusion events are mediated by the assembly of SNARE complexes (fo review Jahn and Scheller 2006). Indeed, NE assembly requires NSF and α-SNAP (Baur et al. 2007), fusion factors that activate SNARE proteins (Jahn and Scheller 2006). Integral membrane GTPases of the ER, called atlastins, were recently found to mediate fusion between ER tubules (Hu et al. 2009; Orso et al. 2009). It will be interesting to see if and when atlastins are involved in fusion events necessary for NE reformation. It is currently unknown whether atlastins and the SNAREs involved in ER fusion, such as syntaxin 18 (Hatsuzawa et al. 2000), act cooperatively to form and maintain the membrane network of the ER or whether they mediate distinct fusion events on different types of membranes. Both machineries mediate the approximation and fusion of ER membranes across a cytoplasmic space (Fig. 3a) and are therefore localized to the cytoplasmic side of the respective membranes or in the cytoplasm. The cytoplasmic membrane fusion events required to re-form the NE should be distinguished from the fusion required for NPC assembly into an intact NE, which occurs during interphase and possibly during post-mitotic nuclear formation (Fig. 3b). The nature and localization of the machinery required for fusion between the inner and ONMs during pore insertion have not been identified but might be non-cytoplasmic.

### Establishing a NE membrane domain

The NE is rapidly established by the concentration of specific proteins from the mitotic ER network on the de-condensing chromatin. In vitro, the recruitment of NE-forming membranes depends on transmembrane proteins (Collas et al. 1996; Newport and Dunphy 1992; Wilson and Newport 1988). Integral proteins of the INM including LBR (Collas et al. 1996; Pyrpasopoulou et al. 1996; Ye and Worman 1994), and the LEM-domain containing proteins Lap2β (Foisner and Gerace 1993; Furukawa et al. 1997), MAN1/LEMD3 (Liu et al. 2003) and emerin (Hirano et al. 2005) bind chromatin. The nucleoplasmic domain of LBR interacts with heterochromatin-binding protein (HP1) (Ye et al. 1997), while the LEM domain-containing proteins interact with the chromatin-associated protein barrier to autointegration factor (BAF) (see Brachner and Foisner 2011). Several proteins of the INM, as well as the transmembrane nucleoporins NDC1 and POM121, also possess intrinsic DNA-binding capacities based on the presence of basic domains (Ulbert et al. 2006b). The use of multiple chromatin interaction strategies by the INM proteins could at least partially account for the rapid accumulation of membranes on chromatin at the onset of anaphase.

With the exception of LBR, for which contradicting results have been reported (Anderson et al. 2009; Lu et al. 2010), none of the INM proteins are essential for nuclear re-assembly in vivo. The depletion of individual INM proteins delays but does not inhibit NE formation in cultured cells and co-depletion of multiple INM proteins or depletion of the chromatin factor BAF, exacerbates the delay (Anderson et al. 2009), suggesting that the chromatin-binding NE proteins could play a redundant role in NE membrane recruitment. Furthermore, removing one INM protein, Lap2β, does not affect the distribution of another, LBR, despite delaying NE formation, implying that the recruitment of various nuclear membrane proteins is not only redundant but also cooperative towards NE assembly.

In addition to chromatin binding by INM proteins, the formation of membrane micro-domains has been proposed to support the segregation of NE membranes from the bulk ER (Mattaj 2004). A number of in vitro experiments in different experimental systems have revealed specific pools of membrane vesicles with the capacity to bind chromatin and give rise to a NE (Antonin et al. 2005; Buendia and Courvalin 1997; Chaudhary and Courvalin 1993; Collas et al. 1996; Ulbert et al. 2006b; Vigers and Lohka 1991; Vollmar et al. 2009). Although these NE membrane populations are likely to originate during the process of their isolation and fractionation when the mitotic ER vesiculates, membrane micro-domains have been found to segregate into distinct vesicles (Simons and Toomre 2000). It is therefore possible that the identification of NE-specific vesicles reflects the existence of micro-domain organization within the seemingly homogeneous mitotic ER.

The existence of NE-specific lipid rafts within the ER is unlikely given the low relative abundance of cholesterol at these membranes. However, the possibility that distinct lipid compositions contribute to functional partitioning at the NE, in analogy to the mitochondria-associated ER membrane (Fujimoto and Hayashi 2011), cannot be excluded. In support of this notion, NE vesicles isolated from sea urchin egg extracts are specifically enriched in phosphoinositides (Larijani et al. 2000), which confer a unique level of fluidity at the membrane (Zhendre et al. 2011). It should be noted that sea urchin pronucleus formation differs significantly from nuclear assembly in vertebrates (Collas 2000) and distinct lipid compositions have not been detected in vertebrate NE membranes to date.

In addition to lipid-mediated domain organization, membrane coating proteins have been proposed to function in micro-domain formation at different endosome compartments (Zerial and McBride 2001). If an analogous strategy is employed by the NE, lamins could represent attractive candidates for the coating protein component. Several INM proteins interact with lamin B (see Wilson and Foisner 2010 for a comprehensive review), which can be found on mitotic ER-derived membrane vesicles (Chaudhary and Courvalin 1993; Gerace and Blobel 1980). However, despite recent advances in the study of membrane micro-domains (Simons and Gerl 2010), there is no direct evidence for NE subdomain formation in the ER, nor is it clear that such domain organization would impact NE reformation.

### Regulating the recruitment of NE membranes to chromatin

Nuclear membranes first re-associate with chromatin during the late stages of anaphase (Daigle et al. 2001; Ellenberg et al. 1997; Robbins and Gonatas 1964). This recruitment can be artificially accelerated in vivo by overexpressing chromatin-binding membrane proteins, or by depleting reticulons to alter ER organization (Anderson et al. 2009). In both cases, premature NE formation interferes with chromosome segregation underlining the importance of robust temporal coordination between chromatin and nuclear membrane dynamics during the cell cycle.

Phosphorylation of nuclear lamins (Heald and McKeon 1990; Peter et al. 1990) and INM proteins (Foisner and Gerace 1993; Pyrpasopoulou et al. 1996) initiates disassembly of the NE at the onset of mitosis. The major driving force of mitotic phosphorylation, cdk1-cyclin B, has been found to inhibit the association of membranes with post-mitotic chromatin in vitro (Newport and Dunphy 1992; Pfaller et al. 1991), likely via one or several downstream kinases (Newport and Dunphy 1992; Vigers and Lohka 1992). If mitotic phosphorylation prevents the association of membranes with chromatin, the process must be reversed at the end of mitosis (Fig. 2, inset). Indeed, membranes isolated from mitotic *Xenopus* egg extracts, containing active cdk1-cyclin B, can be induced to bind chromatin when they are first incubated with interphase cytosol (Ito et al. 2007). This shift in membrane affinity for chromatin is due to the activity of phosphatases, such as PP1 (Ito et al. 2007; Pfaller et al. 1991).

The target of mitotic phosphorylation events that regulate membrane recruitment is on the membranes and not the chromatin (Pfaller et al. 1991). In vitro experiments using protein-free liposomes imply that lipid recruitment to chromatin could be specifically regulated during the cell cycle (Ramos et al. 2006). However, biological membranes are covered with proteins, largely due to mosaics of transmembrane proteins and their interaction partners, with relatively little area of exposed lipids (Dupuy and Engelman 2008; Takamori et al. 2006). Thus although regulation at the lipid surface may be a contributing factor, it is more likely that the cell cycle-dependent recruitment of membranes to chromatin is mediated by the integral nuclear membrane proteins.

Two INM proteins that are recruited quickly following the onset of anaphase, Lapβ and LBR, are phosphorylated during mitosis, preventing their association with chromatin (Foisner and Gerace 1993; Ito et al. 2007; Courvalin et al. 1992). The precise regulation of LBR by mitotic phosphorylation is particularly well studied. In post-mitotic extracts, an arginine-serine repeat-containing region of LBR mediates its recruitment to chromatin (Takano et al. 2002). Phosphorylation of a specific serine residue within this domain prevents LBR binding to chromatin in vitro (Ito et al. 2007; Nikolakaki et al. 1997; Takano et al. 2004) and its de-phosphorylation controls the timing of ER membrane recruitment to anaphase chromosomes in human cells (Tseng and Chen 2011). Given the redundancy of INM protein recruitment, it is likely that other integral NE proteins are regulated similarly. In fact, the pore membrane proteins NDC1, POM121, and GP210 as well as the INM proteins emerin and MAN1 are also phosphorylated during mitosis (Dephoure et al. 2008; Mansfeld et al. 2006; Ellis et al. 1998; Favreau et al. 1996), but the significance of these events with regard to nuclear membrane recruitment is unclear.

Although exit from mitosis is characterized by an overall decrease in phosphorylation, the in vitro association of LBR with chromatin also requires specific phosphorylation events, which are mediated by serine/arginine-rich protein-specific kinase 1 (Nikolakaki et al. 1997; Takano et al. 2002; Dreger et al. 1999). Similarly, Lap2β is phosphorylated within its chromatin-binding region during interphase (Dreger et al. 1999). These observations suggest that a simple model of mitotic phosphorylation and post-mitotic de-phosphorylation cannot account for the precise timing of membrane recruitment to chromatin but rather that multiple site-specific phosphorylation events tune this process.

In addition to cell cycle-dependent phosphorylation events, transport receptors and the GTPase ran may regulate the association of INM proteins with chromatin. Chromatin is demarcated throughout the cell cycle by a high concentration of the GTP-bound ran (Kalab et al. 2002). Ran is best known for its function in nucleo-cytoplasmic transport across the NPC, where it stimulates the release of importin-bound cargo in the nucleus, but it is also required for nuclear assembly in vitro (Hetzer et al. 2000; Zhang and Clarke 2000), where it provides the positional information necessary to specify that nuclear assembly occurs on the de-condensing chromatin (for review, see Hetzer et al. 2002). Integral membrane proteins can be targeted to the interphase NE via importins (Doucet et al. 2010; Turgay et al. 2010), and it is possible that the ran-importin system could similarly regulate the recruitment of INM proteins to post-mitotic chromatin (Turgay et al. 2010; Antonin et al. 2011) (Fig. 2, inset). In agreement with this notion, LBR was found to interact with importin β during mitosis (Ma et al. 2007; Lu et al. 2010) and this inhibitory complex could be dissociated in the presence of ranGTP (Ma et al. 2007). The importin β and chromatin-binding sites of LBR overlap, thus it is conceivable that members of the importin family act as molecular chaperones to prevent undesired interactions between the DNA-binding domains of INM proteins and chromatin during mitosis.

It has also been suggested that importins mediate the recruitment of NE membranes to chromatin by bridging the membrane precursors and either ran (Ma et al. 2007) or NLS-containing chromatin proteins (Lu et al. 2012). This model requires a stable interaction between ran and importins, which is difficult to rectify with the ran-dependent dissociation of importin-cargo complex during nuclear import. Furthermore, it is not clear how a non-canonical importin interaction with two binding partners is established. Nonetheless, the contribution of NLS-containing chromatin proteins could represent an important link between post-mitotic chromatin structure and membrane recruitment and warrants further investigation.

In summary, the timing of nuclear membrane recruitment to chromatin is regulated by the reversal of mitosis-specific phosphorylation events on nuclear membrane proteins. With few exceptions, the relevant target proteins, precise sites of modification, and the phosphatases involved have yet to be identified. Spatial organization by the ran system might contribute to nuclear membrane recruitment by exposing DNA-binding domains of membrane proteins in the vicinity of chromatin but the relevance of such a mechanism has not been determined.

### Building NPCs in the NE: post-mitotic assembly modes

As the ER membranes are reorganized to accommodate the distinct composition of the NE and enclose the chromatin, the coordinated assembly of NPCs begins. NPCs form large pores in the NE, having a diameter of approximately 100 nm. Unlike membrane transporters, which give rise to channels within a single lipid bilayer, NPCs span two lipid bilayers at sites where the outer and inner membranes of the NE are fused. As a result, only a small sub-fraction of the roughly thirty NPC proteins (nucleoporins) are integral membrane proteins residing in the pore membrane. Most nucleoporins do not possess membrane-spanning domains and are thus recruited from the cytosol to assemble NPCs at the conclusion of open mitosis in animals.

**Fig. 4:**
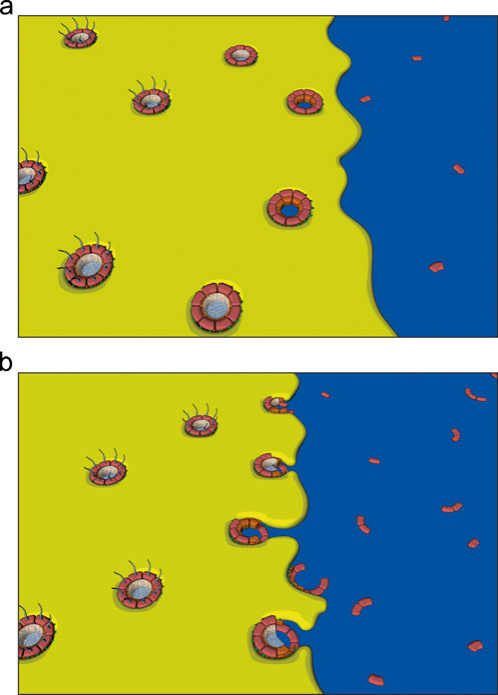
Post-mitotic NPC assembly as envisioned by the insertion and enclosure models. As the cisternal sheets of the nuclear membrane wrap around chromatin, NPC assembly proceeds by either insertion into the locally intact nuclear envelope (**a**) or by enclosure of NPC assembly intermediates by the outgrowing membranes (**b**). In both cases, NPC assembly is initiated by the Mel-28/ELYS-dependent recruitment of the Nup107-160 complex to chromatin. Following the initial contact between nuclear membranes and the Nup107-160 complex, additional nucleoporins are incorporated in the assembling NPCs (see also Fig. 5 for details)

Post-mitotic NPC assembly has been proposed to proceed via two fundamentally different modes: insertion or enclosure. In an insertion model (Fig. 4a), NPCs assemble and integrate into the two juxtaposed membrane sheets of the intact NE (Fichtman et al. 2010; Lu et al. 2011; Macaulay and Forbes 1996). Formation of the pore requires the fusion of the outer and INMs across the lumen of the NE (see Fig. 3b). In dividing metazoan cells the number of NPCs roughly doubles during interphase (Dultz and Ellenberg 2010; Maul et al. 1972; Doucet and Hetzer 2010), when NPCs must be formed by insertion into the intact NE. Furthermore, organisms that employ closed mitosis for cell division, such as yeast, can only assemble new NPCs by insertion into the intact nuclear membranes (Rexach 2009; Winey et al. 1997). Thus, an insertion model represents a unifying mechanism for NPC assembly across species and in all stages of the cell cycle.

In contrast to interphase NPC assembly, which occurs as a collection of singular and sporadic events, the post-mitotic assembly of thousands of NPCs in metazoan cells proceeds simultaneously and rapidly, on average one order of magnitude faster, in order to quickly re-establish nuclear compartmentalization (Dultz and Ellenberg 2010; D’Angelo et al. 2006; Dultz et al. 2008). The distinct kinetics of post-mitotic NPC formation could be explained by the use of a mechanistically unique assembly mode. Enclosure models suggest (Antonin et al. 2008; Burke and Ellenberg 2002; Walther et al. 2003a) that post-mitotic NPC assembly following open mitosis does not occur by insertion into intact membrane sheets but is rather completed by the envelopment of the assembling NPCs on the chromatin surface by the outgrowing ER-derived membranes (Fig. 4b). In this case, NPC assembly is initiated by the recruitment of the Nup107-160 complex to chromatin, which has been observed in vitro (Walther et al. 2003a) and in vivo (Dultz et al. 2008; Belgareh et al. 2001). Membranes are subsequently recruited, resulting in the enrichment of NE-specific membrane proteins, including the integral membrane nucleoporins POM121 and NDC1 (Antonin et al. 2005; Mansfeld et al. 2006; Rasala et al. 2008). Kinetic analyses of individual NPC proteins suggest that the ordered recruitment of NE components at the end of mitosis is distinct from interphase pore assembly, where POM121 gradually accumulates prior to the recruitment of the Nup107-160 complex (Doucet et al. 2010; Dultz and Ellenberg 2010). This reversal of recruitment events implies that post-mitotic NPC assembly is initiated on the chromatin, as the enclosure model proposes, while interphase insertion of NPCs begins on the nuclear membranes.

The DNA-binding protein Mel-28/ELYS recruits the Nup107-160 complex and acts as a seeding point for post-mitotic NPC assembly (Franz et al. 2007; Gillespie et al. 2007; Rasala et al. 2008). In agreement with the notion of chromatin-directed NPC assembly at the end of mitosis, Mel-28/ELYS is essential for post-mitotic NPC formation but is dispensable to this end during interphase (Doucet et al. 2010). Conversely, the transmembrane nucleoporin POM121 is proposed to be specifically required for interphase NPC assembly, where it initiates pore formation on the membranes. However, it should be noted that the dispensability of POM121 for the formation of NPCs at the end of mitosis (Doucet et al. 2010) is not a consistent observation in the field (Antonin et al. 2005; Shaulov et al. 2011) and could be attributed to an incomplete depletion, resulting in a small pool of residual POM121 that was sufficient for post-mitotic assembly but completely consumed when nuclei reached interphase.

The existence of membrane intermediates specific to post-mitotic and interphase pore formation can be inferred from differences in the requirement of membrane bending and curvature-sensing proteins. Our recent work demonstrates distinct functions of the membrane-associated nucleoporin Nup53, which are essential for pore formation post-mitotically or during interphase (Vollmer et al. 2012). While either of the two Nup53 membrane-binding sites is sufficient for post-mitotic NPC assembly, interphase assembly specifically requires the second binding site at the C terminus. As the C-terminal-binding site was also found to induce membrane curvature, this could indicate that a unique membrane deformation activity is required for interphase pore assembly. Similarly, ER-bending proteins of the reticulon family that induce convex membrane curvature (Hu et al. 2008) were found to be important for NPC assembly into the intact NE both in yeast and vertebrates (Dawson et al. 2009). It is difficult to ascertain whether reticulons also contribute to post-mitotic NPC assembly because their role in ER membrane reorganization at the end of mitosis is a prerequisite for NE reformation (Anderson and Hetzer 2008a). Interestingly, a membrane curvature sensing domain of the Nup107-160 complex member Nup133 was found to be required for interphase but not post-mitotic assembly (Doucet and Hetzer 2010). It is possible that specific membrane curvature events are required during the insertion of interphase NPCs when the two nuclear membranes approximate and fuse (Fig. 3b). Other modes of pore membrane stabilization might be sufficient at the end of mitosis, when NPCs on the chromatin are enclosed by the outgrowing ER.

The existence of cell cycle-dependent differences in the molecular requirements of NPC formation does not unambiguously prove the use of distinct assembly mechanisms. The specific requirement for Mel-28/ELYS during post-mitotic assembly, for example, could rather reflect a need for the efficient recruitment of NPC components during open mitosis when they cannot be enriched in the nucleus by active NPC-dependent import. Assembly of NPCs into an intact envelope requires the Nup107-160 complex on the nucleoplasmic site of the NE (D’Angelo et al. 2006; Walther et al. 2003a). Thus, regardless of the assembly mode employed, NPC components will need to be enriched on the chromatin at the end of mitosis. Similarly, the unique requirement for proteins inducing membrane curvature during interphase NPC formation does not prove the use of dissimilar assembly mechanisms at different points in the cell cycle although it strongly implies distinct modes.

Nuclear formation can be decelerated in *Xenopus* extracts, which are commonly used to recapitulate post-mitotic NPC assembly, by reducing the temperature of the reaction (Fichtman et al. 2010). Under these conditions, a NE intermediate that possesses a closed NE but no pores or NPCs can be observed, suggesting that post-mitotic NPC assembly proceeds by insertion and requires the fusion of outer and INMs. However, the lower temperature might specifically inhibit or delay the post-mitotic mode of assembly, resulting in an artificial bias towards interphase NPC assembly. Recent live cell imaging experiments suggest that the local generation of NE membranes on chromatin from ER cisternae precedes NPC assembly, which would also implicate an insertion mode for post-mitotic NPC assembly (Lu et al. 2011). However, the precise order of recruitment, particularly with regard to the small number of nucleoporins that might be sufficient to seed NPC assembly is difficult to ascertain. In order to ultimately resolve this issue, it will be crucial to determine whether the hitherto unknown factors mediating fusion of the outer and INM are equally required for interphase and post-mitotic NPC assembly.

Importantly, while the tubular or cisternal organization of the post-mitotic ER recruited to chromatin would appear to favor enclosure or insertion of NPCs, respectively, these structures are in principle compatible with both assembly modes. Although it is easy to imagine how intermediates of NPC assembly are seeded in the gaps of a tubular ER network and enclosed by the flattening and expansion of those membrane areas (Anderson and Hetzer 2008b; Antonin et al. 2008), an ER network on the chromatin surface might first close the gaps to form a closed NE into which NPCs are assembled according to the insertion model. Similarly, outgrowth of flat ER cisternae could first form a closed NE, at least locally, into which NPCs are inserted (Lu et al. 2011) (Fig. 4a). However, it is also possible that the growing sheets of the cisternae enclose assembling NPC intermediates similar to waves flowing around wooden posts on a beach (Fig. 4b).

### Assembling NPCs at the end of mitosis: ordered recruitment of nucleoporins

**Fig. 5:**
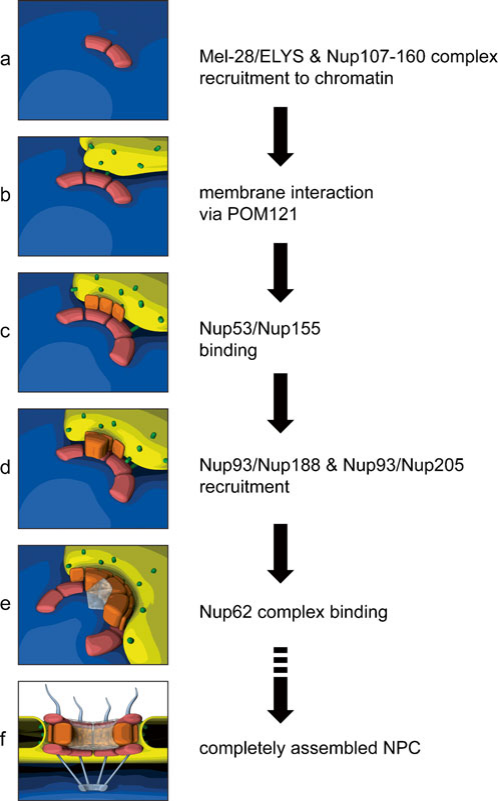
Model for the ordered assembly of NPCs at the end of mitosis (see text for details and alternative models). The DNA-binding nucleoporin Mel-28/ELYS initiates NPC assembly on the chromatin by recruiting the Nup107-160 complex (**a**), which in turn associates with the nuclear envelope membranes via the transmembrane nucleoporin POM121 (**b**). The recruitment of the Nup93 complex is mediated by its membrane-associated nucleoporins, Nup53 and Nup155, which interact with integral membrane proteins at the nascent pore membrane (**c**) and promote the incorporation of Nup93, Nup188, and Nup205 to complete the structural backbone of the NPC (**d**). The subsequent recruitment of FG-repeat containing nucleoporins of the Nup62 complex (**e**) combined with the previous association Nup98 (not shown) establishes the central channel, a hydrophobic meshwork that confers the transport properties of the NPC. The fully assembled NPC (**f**) consists of multiple copies of the component nucleoporins, which are arranged in octagonal symmetry to create a cylindrical channel. Peripheral structures include the cytoplasmic filaments and the nuclear basket, protruding from opposite faces of the NPC. Initial membrane contact (**b**) is depicted according to the enclosure model. It should be noted that the order of events is the same for both the enclosure and insertion modes of NPC assembly

A single vertebrate NPC has a mass of roughly 60MDa, an approximate diameter of 100 nm and consists of multiple copies of 30 unique nucleoporins, which are arranged to give rise to a cylindrical pore with eightfold rotational symmetry (Brohawn et al. 2009). Nucleoporins can be categorized based on their contribution to either the structural scaffold or the transport properties of the NPC. The latter group consists of nucleoporins with phenylalanine-glycine (FG) repeat sequences that mostly occupy the central channel of the pore and contribute to the diffusion barrier and regulated transport capacity of the NPC (Weis 2007). The construction of this macromolecular structure is accomplished by the sequential recruitment of nucleoporins (Bodoor et al. 1999; Dultz et al. 2008; Haraguchi et al. 2000). Immunofluorescence and live cell imaging in cultured mammalian cells, as well as depletion experiments in *Xenopus* egg extracts, have elucidated the order and interdependence of the important recruitment steps (Fig. 5).

Post-mitotic NPC assembly starts on chromatin, where the DNA-binding nucleoporin Mel-28/ELYS recruits the Nup107-160 complex (Franz et al. 2007; Walther et al. 2003a; Rotem et al. 2009; Rasala et al. 2008; Harel et al. 2003). In vitro, these events can occur in the absence of membranes. The subsequent association of the transmembrane nucleoporin POM121 at the newly forming pores (Antonin et al. 2005) is thought to be mediated by direct binding of POM121 to the Nup107-160 complex (Mitchell et al. 2010; Yavuz et al. 2010) and constitutes the first connection between the assembling NPC and nuclear membranes. The transmembrane nucleoporin NDC1 is also found at the NE around this time. The steps following membrane recruitment can be ordered in space starting from the membrane and building laterally towards the center of the pore, as was suggested by the protein-protein interaction network of yeast (Rexach 2009). First, the Nup93 complex joins the assembling pore (Dultz et al. 2008). Recent experiments in *Xenopus* egg extracts suggest that the recruitment of the Nup93 complex proceeds by assembly of the individual components rather than by recruitment of the pre-assembled complex (Sachdev et al. 2012; Theerthagiri et al. 2010; Vollmer et al. 2012). Of these components, Nup53 is the first to associate with the nascent pore, probably followed by Nup155. Both proteins interact with the transmembrane nucleoporins NDC1 and POM121 (Mansfeld et al. 2006; Mitchell et al. 2010; Yavuz et al. 2010) and thus provide a second link between the NPC and membranes at the pore. The capacity for Nup53 to interact directly with membranes may further contribute to the formation or stability of the growing NPC (Vollmer et al. 2012). Nup93 interacts with Nup53 and is consequently incorporated, along with its binding partners Nup188 and Nup205 to complete the structural backbone of the pore. Nup93 subsequently recruits the FG repeat-containing nucleoporins of the Nup62 complex. The FG-containing nucleoporin Nup98 is recruited concomitantly with Nup93 (Dultz et al. 2008) and has recently been found to be key to the establishment of the transport and exclusion properties of the pore (Hulsmann et al. 2012; Laurell et al. 2011). Together, these FG nucleoporins form a substantial part of the hydrophobic meshwork in the center of the pore (Ribbeck and Gorlich 2001).

Open questions remain regarding the construction of a fully assembled NPC. Many nucleoporins, including the Nup107-160 complex, are symmetrically distributed on both the nucleoplasmic and cytoplasmic side of the NPC (Brohawn et al. 2009; Rout et al. 2000; Belgareh et al. 2001) but the timing and mechanistic details regarding assembly of the cytoplasmic portion of the NPC are largely unknown. Similarly, the formation of peripheral NPC structures, such as the nuclear basket and the cytoplasmic filaments, follows the establishment of the structural pore and central channel but the precise order of events is not well defined. Finally, although the complete pore possesses octagonal symmetry, it is not clear whether the numerous copies of each subcomplex are recruited simultaneously. This question is beyond the resolution of current experimental techniques.

### Regulating NPC assembly on chromatin at the end of mitosis

Nucleoporins play diverse roles during mitosis (for review, see Chatel and Fahrenkrog 2011) but they do not assemble NPCs until mitotic exit. Multiple nucleoporins, including members of the Nup107-160 complex, Nup98, and Nup53, are phosphorylated by mitotic kinases (Favreau et al. 1996; Glavy et al. 2007; Laurell et al. 2011; Macaulay et al. 1995; Mansfeld et al. 2006; Onischenko et al. 2005), and it is tempting to speculate that mitotic phosphorylation acts as a general mechanism to keep nucleoporins in a dissociated state. Indeed, hyperphosphorylation of Nup98 interferes with its associations at the pore and initiates the disassembly of the NPC at the start of mitosis (Laurell et al. 2011). Conversely, dephosphorylation at the end of mitosis should promote interactions between nucleoporins and thus NPC assembly. In most instances direct evidence for such a mechanism is lacking because the kinases and phosphatases responsible perform a plethora of functions that are essential to mitotic entry, progression, and exit. Furthermore, the identification of decisive phosphorylation events is complicated by a high degree of redundancy. For example, Nup98 is phosphorylated at 13 different sites by cdk1 and members of the NIMA-related kinase family during mitosis (Laurell et al. 2011).

Spatial regulation of NPC assembly on chromatin is provided by the localized concentration of ranGTP (Kalab et al. 2002). The importance of this spatial information is underlined by the aberrant formation of NPCs in ER membrane stacks apart from NE when the ranGTP gradient is disturbed (Walther et al. 2003b). Transport receptors of the importin family bind a large proportion of nucleoporins and have been proposed to regulate the post-mitotic formation of NPCs by blocking the relevant interactions between NPC components (Harel et al. 2003; Walther et al. 2003b). This inhibition is reversed by the ranGTP-dependent release of importin-bound nucleoporins in the vicinity of chromatin, which is required for NPC formation at the NE. MEL-28/ELYS and the Nup107-160 complex represent attractive candidates for such a mode of regulation because they bind transport receptors and associate with chromatin in the early stages of NPC assembly (Walther et al. 2003b; Rasala et al. 2008; Rotem et al. 2009; Franz et al. 2007). However, the functional outcome of transport receptor binding is generally difficult to dissect due to the existence of multiple distinct interaction-dependent activities. Interactions between FG nucleoporins and transportins or importins are required to facilitate transport of cargoes through the NPC, a function that may also extend to other NPC components, such as Nup50 (Lindsay et al. 2002). Several nucleoporins also bind transport receptors in order to be imported to the nucleoplasmic side of the pore, where they contribute to interphase NPC assembly. The integral membrane nucleoporin POM121 is transported in this way (Doucet et al. 2010; Funakoshi et al. 2011) and it is likely that the Nup107-160 complex employs a similar mechanism. Thus, as for the temporal regulation of NPC assembly, challenges still lie ahead in deciphering the molecular mechanisms that control NPC assembly on post-mitotic chromatin.

### Unpacking chromatin during mitotic exit

In metazoans, the establishment of an interphase nucleus that is competent for regulated transcription and replication depends on the coordination of chromatin de-condensation and NE formation during mitotic exit. Whereas the molecular mechanisms involved in NE and NPC assembly are beginning to emerge, much less is known about the contemporaneous changes that occur on chromatin at the end of mitosis.

We are only starting to understand the molecular and structural dynamics that determine chromatin organization. This reflects the inherent complications in investigating the complexity of protein-DNA interactions involved in packing DNA molecules into chromatin (Maeshima et al. 2010). As a result, the structural rearrangements and relevant effector molecules that enable the 50-fold compaction of mammalian mitotic chromosomes are far from understood (for review, see Belmont 2006; Ohta et al. 2011).

Chromosomes achieve maximal compaction during anaphase, a feat that requires the chromokinesin KID (Ohsugi et al. 2008) and the mitotic kinase Aurora B at the chromatin (Mora-Bermudez et al. 2007). The de-condensation of mitotic chromosomes during late anaphase of mitosis requires the extraction of polyubiquitinylated Aurora B by the AAA ATPase p97 (Ramadan et al. 2007). The precise consequences of Aurora B inhibition, including the relevant targets of this kinase, and of other p97-dependent activities are not currently understood. In addition to p97, the protein phosphatase PP1 and its nuclear targeting unit PNUTS have been implicated in post-mitotic chromatin de-condensation (Landsverk et al. 2005; Lee et al. 2010). Mitotic exit is generally promoted by the activities of PP1 and PP2A (Wurzenberger and Gerlich 2011) but the molecular targets regulated by these phosphatases, particularly with regard to post-mitotic changes to chromatin structure, are largely unknown.

### Coordinating the establishment of the NE and interphase chromatin architecture

Interphase chromatin architecture is not random. Individual chromosomes occupy distinct territories within the 3D organization of the nucleus, which are maintained throughout a lifetime of cell divisions (reviewed in Cremer et al. 2006; Misteli 2007). Furthermore, highly condensed chromatin regions, known as heterochromatin, are predominantly found at the nuclear periphery and have been observed in close proximity to the NE (reviewed in Akhtar and Gasser 2007; Francastel et al. 2000). Proteins of the nuclear lamina, INM, and NPC interact with chromatin during interphase and have been implicated in chromatin organization at the envelope. Our current understanding of how these interactions impact chromatin structure and transcriptional activity are beyond the scope of this review and are discussed thoroughly by others (Capelson et al. 2010; Zuleger et al. 2011; Kubben et al. 2010).

Although many of the interactions between chromatin and proteins at the nuclear periphery are either transient or established following the formation of a closed NE, the broad organization of chromatin in the nucleus must be established as the chromosomes de-condense and could be coupled to NE formation. In support of this hypothesis, common focal points for chromatin condensation and de-condensation have been observed at the nuclear periphery (Hiraoka et al. 1989). Multiple transmembrane proteins of the NE have been found to impact chromatin de-condensation (Korfali et al. 2010; Chi et al. 2007). However, the molecular mechanisms that account for the involvement of NE proteins in chromatin de-condensation have yet to be elucidated.

We are therefore still confronted with the question of how chromatin organization is established at the end of mitosis. A recent study suggests that the peripheral identity of chromatin is maintained throughout the cell cycle (Olins et al. 2011). Peripheral chromatin of both interphase nuclei and mitotic chromosomes, termed epichromatin, can be characterized by presence of a specific nucleosome-based and conformation-dependent epitope. Although the functional significance of epichromatin is currently unclear, it is tempting to speculate that the continuity of peripheral chromatin architecture contributes to the establishment of nuclear organization at the end of mitosis. In this context, epichromatin could provide a scaffold for components of the NE. Phosphatidylserine is associated with histones specifically localized to epichromatin and it might provide a seeding point for nuclear membranes at these defined chromatin regions (Prudovsky et al. 2012). During mitosis, a layer of largely nucleoplasmic proteins and ribonucleoproteins, collectively referred to as perichromatin, associates with non-repetitive DNA sequences at the chromatin periphery (for review, see Hernandez-Verdun and Gautier 1994; Van Hooser et al. 2005), and it is possible that epichromatin also mediates this localization. Importantly, perichromosomal components have been proposed to contribute to the early events of post-mitotic nuclear assembly (Hernandez-Verdun and Gautier 1994).

Structural features of chromatin are often correlated with post-translational modifications to histones. As specific histone phosphorylation and methylation events are reportedly coordinated with the cell cycle (Oki et al. 2007; Markaki et al. 2009), they might contribute to the changes in chromatin structure observed during the cell cycle. However, there is no evidence that histone modifications actually mediate the dramatically altered compaction of chromatin during mitosis. For example, histone H3 phosphorylation at serine 10 is perhaps the most prominent mitotic histone modification but it is not essential for chromatin condensation in yeast or vertebrates (Hsu et al. 2000; MacCallum et al. 2002). Nonetheless, cell cycle-specific histone modifications could regulate the association of non-histone factors with chromatin.

Two chromatin-binding proteins, HP1 and BAF, provide a link between the de-condensing chromosomes and NE assembly by binding to LBR and LEM domain containing INM proteins, respectively. Interestingly, HP1 binding to chromatin is inhibited by H3 phosphorylation at serine 10 and promoted by methylation at lysine 9 (Fischle et al. 2005; Bannister et al. 2001; Lachner et al. 2001; Hirota et al. 2005). Although LBR can interact directly with histones and with other chromatin-associated proteins (reviewed in Olins et al. 2010), the regulation of HP1 binding by cell cycle-dependent modifications of histone H3 could regulate the post-mitotic association of the INM protein with chromatin. BAF binds to both histone H3 and histone H1 in vitro, which might mediate its interaction with chromatin, but this interaction is not dependent on post-translational histone modifications (Montes de Oca et al. 2005). Instead, BAF has been found to promote the accumulation of interphase histone H3 marks at the end of mitosis (Montes de Oca et al. 2011).

The recruitment of BAF to chromatin occurs in early anaphase and is required for post-mitotic NE assembly (Gorjanacz et al. 2007; Margalit et al. 2005; Segura-Totten et al. 2002; Furukawa et al. 2003). BAF directs the post-mitotic incorporation and interphase distribution of LEM-domain containing proteins, which reciprocally modulate the distribution of BAF during interphase (Haraguchi et al. 2008; Margalit et al. 2007; Ulbert et al. 2006a; Brachner and Foisner 2011). The INM protein LEM4 was recently found to act at the convergence of NE assembly and chromatin structure (Asencio et al. 2012). During mitosis, the association of BAF with chromatin is negatively regulated by vrk1-dependent phosphorylation (Gorjanacz et al. 2007; Nichols et al. 2006), which is reversed by PP2A upon mitotic exit (Asencio et al. 2012). These counteracting events require LEM4 and its interaction with both the kinase and the phosphatase to control BAF-dependent NE assembly on the chromatin. It is currently unclear how the interaction of LEM4 with vrk1 and PP2A is controlled to ensure this regulation. As LEM2 was found to interact with PP1, it will be interesting to determine whether a similar regulatory mechanism is employed and to identify the downstream targets. PP1 has been implicated as a link between chromatin re-organization during mitotic exit and NE reassembly with its regulatory subunit RepoMan (Vagnarelli et al. 2011).

Fluorescence imaging data from human cells indicates that DNA-binding and INM proteins are not recruited uniformly to chromatin at the end of mitosis. From late anaphase until the establishment of an import competent nucleus, the chromatin mass can be divided into two distinct territories. When telophase chromatin is viewed in the axis of the mitotic spindle, the “core” refers the central region at surfaces both proximal and distal to the spindle. Along the same axis, the more peripheral chromatin domain corresponds to “noncore” chromatin. The core region is enriched in A-type lamins and is established by the local accumulation of BAF (Haraguchi et al. 2008). Accordingly, Lap2β and emerin are found at core chromatin in late anaphase before being distributed rather homogeneously at the rim of the completed nucleus (Dabauvalle et al. 1999; Haraguchi et al. 2000). Conversely, LBR is recruited to the noncore region, where nucleoporins and lamin B also accumulate (Chaudhary and Courvalin 1993; Haraguchi et al. 2000, 2008). The DNA-binding nucleoporin MEL-28/ELYS was recently found to control the establishment of these subdomains (Clever et al. 2012), an event that requires the Nup107-160 complex. Thus, the initial stages of NPC formation on chromatin are linked to the establishment of distinct chromatin regions. The importance of these transient chromatin domains in the establishment of a functional nucleus has yet to be determined.

## Conclusions

Re-establishing the vertebrate nuclear compartment after mitosis invokes remarkable changes to chromatin structure and ER membrane organization. As chromatin de-condenses, NPCs are assembled and incorporated in the re-forming nuclear membranes to ensure that regulated exchange can occur across the otherwise impermeable nuclear boundary. The construction of a functional nucleus thus requires seamless coordination and multifaceted interactions between membrane, NPC, and chromatin components. Nuclear membranes are segregated from the mitotic ER in anaphase due to interactions between transmembrane proteins destined for the NE with chromatin-associated factors. Assembly of copious NPCs is also initiated on chromatin, but whether NPCs assemble and are inserted into intact NE sheets or are rather enclosed by the re-forming NE remains controversial. Mitotic kinases and phosphatases, along with the activity of the ran system, provide the temporal and spatial cues that control nuclear membrane and NPC protein recruitment and assembly on de-condensing chromatin. The molecular mechanisms underlying the transition of nuclear membranes and NPC components to a post-mitotic state with the capacity to form a NE are beginning to emerge. Comparatively, little is known about the series of structural changes that occur on chromatin during de-condensation and render it competent for the initial recruitment of nuclear membranes and NPC components. The faithful completion of post-mitotic nuclear assembly relies on the coordination of major NE and chromatin restructuring events as well as the construction of functional NPCs, and it is likely that several mitotic signaling nodes link these processes.
